# Association of gestational diabetes mellitus diagnosed at different time points in oral glucose tolerance test with adverse pregnancy outcomes: a retrospective cohort study

**DOI:** 10.3389/fendo.2025.1493520

**Published:** 2025-05-01

**Authors:** Ruipeng Lin, Yulong Zhang, Yuxin Lin, Lili Yang, Jiayi Chen, Qingxiu Li, Haibo Li, Qian Zhang

**Affiliations:** ^1^ Department of Epidemiology and Health Statistics, School of Public Health, Fujian Medical University, Fuzhou, Fujian, China; ^2^ Department of Obstetrics and Gynecology, Fujian Maternity and Child Health Hospital, College of Clinical Medicine for Obstetrics & Gynecology and Pediatrics, Fujian Medical University, Fuzhou, Fujian, China; ^3^ Division of Birth Cohort Study, Fujian Maternity and Child Health Hospital, College of Clinical Medicine for Obstetrics & Gynecology and Pediatrics, Fujian Medical University, Fuzhou, Fujian, China

**Keywords:** gestational diabetes mellitus, plasma glucose, adverse pregnancy outcomes, oral glucose tolerance test, impaired fasting glucose, impaired glucose tolerance

## Abstract

**Background:**

This study aims to explore the association between gestational diabetes mellitus (GDM) diagnosed at different time points in the oral glucose tolerance test (OGTT) and adverse pregnancy outcomes (APO).

**Methods:**

A retrospective cohort study based on the 75g OGTT conducted in Fujian Maternity and Child Health Hospital. GDM was diagnosed if plasma glucose levels exceeded the threshold at any time point (5.1 mmol/L at 0h, 10.0 mmol/L at 1h, and 8.5 mmol/L at 2h). Binary logistic regression and subgroup analysis were used to analyze the association between abnormal plasma glucose in OGTT and APO.

**Results:**

The study included 37,598 normal pregnancies and 11,302 APO. Compared to the normal group, pregnant women with GDM and abnormal plasma glucose at different time points had an increased risk of APO. Group 2 (abnormal at 0h, but normal at 1h and 2h), Group 3 (normal at 0h, but abnormal at 1h or 2h), and Group 4 (abnormal at 0h, 1h or 2h) showed an increasing trend in APO risk compared to Group 1 (normal at three time points), with adjusted OR of 1.14, 1.18, and 1.42, respectively (*P*<0.001). The subgroup analysis showed no statistically interaction, and the sensitivity analysis results were stable.

**Conclusion:**

Abnormal plasma glucose at different time points is associated with the risk of APO, with the highest risk observed in those with abnormalities at all time points. Future health management for high-risk pregnant women should be strengthened by considering abnormal plasma glucose at different time points.

## 1 Introduction

Gestational diabetes mellitus (GDM) is medically defined as an abnormal glucose tolerance condition that arises or is initially diagnosed during pregnancy. GDM is among the most frequently encountered metabolic complications during pregnancy (1). A meta-analysis conducted in 2019 revealed that the incidence of GDM in China was 14.8% (95%CI: 12.8-16.7%) (2). Over the past decades, the prevalence of GDM has gradually escalated, with projections indicating a further increase. This trend poses a considerable threat to public health in China, particularly given the increasing burden of chronic non-communicable diseases among mothers and their fetuses (3).

GDM is associated with adverse pregnancy outcomes (APO) and long-term complications for pregnant women (4, 5). Our previous study found that elevated plasma glucose at different time points of OGTT was associated with congenital heart disease in fetuses (6). The damage to the free radical elimination mechanism in pregnant women with GDM may expose the fetuses to the harmful effects of oxidative stress, increasing the probability of congenital malformations in fetuses (7). A study has also shown that GDM may increase the risk of preterm premature rupture of membranes (RR=2.34, 95%CI: 1.16-4.69) (8). Maternal hyperglycemia stimulates an increase in fetal insulin production and fat storage, leading to macrosomia (9, 10). These studies suggest that elevated plasma glucose is a risk factor during pregnancy. Pregnancy normally induces insulin resistance, which can also occur in women with GDM (11). According to Ferrannini’s research (12), the mechanism underlying insulin resistance caused by impaired fasting glucose (IFG) differs from that caused by impaired glucose tolerance (IGT). IFG reflects the effect of basal insulin during the night, necessitating intensified insulin therapy, whereas IGT represents glucose metabolism after eating, which can be managed through diet and exercise (13). As research has progressed, IFG and IGT have been identified as the criteria for diagnosing prediabetes (14). Utilizing OGTT at different time points provides a more comprehensive depiction of a pregnant woman’s plasma glucose levels. However, the associations between OGTT 0h, 1h, 2h plasma glucose and APO remains unclear.

Therefore, we conducted a retrospective cohort study based on hospitals, focusing on investigating the association between GDM diagnosed at different time points in the oral glucose tolerance test (OGTT) and the risk of APO.

## 2 Materials and methods

### 2.1 Study population and data collection

This study was based on the Fujian Maternal and Child Health Hospital and adopted a retrospective cohort method. Data were collected from the National Newborn Network from January 2014 to December 2020. Information collected included maternal age, infant sex, delivery mode, gravidity, parity, history of assisted reproduction, history of gestational hypertension, history of gestational thyroid disorders, OGTT results and birth outcomes. Exclusion criteria included pregestational diabetes mellitus, gestational weeks<24 and >40, multiple pregnancies, fetal malformation and abortion outcomes. Participants were informed of the purpose of data collection, the collection and transmission of data were approved by the Ethics Committee of Fujian Maternity and Child Health Hospital (2021KR023), all methods were performed in accordance with the relevant guidelines and regulations.

### 2.2 Main exposure

According to the standards of the International Association of Diabetes and Pregnancy Study Groups (IADPSG), the 75g OGTT is conducted during the 24th to 28th week of gestation (15). For the three days before the test, pregnant women should maintain a normal diet, ensuring that their daily carbohydrate intake is no less than 150g. They should avoid using medications that may affect plasma glucose levels and fast for at least 8h before the test. An intravenous blood sample was taken on the morning of the trial, followed by the intake of 300ml solution containing 75g glucose within 5min. Next, plasma was taken again 1h and 2h later and glucose oxidase was used to measure plasma glucose levels. Three measurements should be below 5.1mmol/L, 10.0mmol/L and 8.5mmol/L (or 92mg/dL, 180mg/dL and 153mg/dL), respectively. If plasma glucose levels in any one measurement meet or exceed these thresholds, GDM is diagnosed.

### 2.3 Outcome definition

Fetal pregnancy outcomes are determined by collecting follow-up data on gestational weeks, birth weight and height of neonates. In this study, APO are defined as preterm birth (PTB), low birth weight (LBW), macrosomia, small for gestational age (SGA) and large for gestational age (LGA). PTB refers to neonates born before 37 weeks of gestation; LBW refers to neonates with a birth weight<2,500g; macrosomia refers to neonates with a birth weight≥4,000g; SGA refers to neonates with a birth weight below the 10th percentile of the average weight for their gestational age; and LGA refers to neonates with a birth weight above the 90th percentile of the average weight for their gestational age (16).

### 2.4 Statistical analysis

For continuous variables, such as age, we used the mean ± standard deviation and the Kruskal-Wallis rank sum test to evaluate the differences in between-group comparisons. For categorical variables, we used frequency (percentage) and the Chi-square test to compare the differences between groups. Based on the plasma glucose at 0h, 1h and 2h of the OGTT, the participants were divided into four groups: Group 1 represented subjects with normal plasma glucose at three time points (0h, 1h and 2h). Group 2 represented subjects with abnormal plasma glucose at 0h, but normal levels at 1h and 2h. Group 3 represented subjects with normal plasma glucose at 0h, but abnormal levels at 1h or 2h. Group 4 represented subjects with abnormal plasma glucose at 0h, 1h or 2h. Binary logistic regression was used to assess the association between a single covariate and APO, and further adjustments for confounding factors were made to evaluate the associations between GDM, abnormal plasma glucose at different time points, and APO risk. Trend tests were conducted using logistic regression based on continuous variables. Subgroup and sensitivity analyses were also conducted to verify the stability of the results. Subgroup analyses were used for elderly parturient, assisted reproduction, delivery mode, infant sex, and gestational hypertension, with likelihood ratio tests used to explore potential interactions between subgroups. Sensitivity analyses were conducted using logistic regression to compare outcomes after excluding high-risk populations (elderly parturient, assisted reproduction, gestational thyroid disorders, and gestational hypertension) with analyses retaining these groups All analyses are conducted using R version 4.3.3, and *P*<0.05 is considered statistically significant.

## 3 Results

### 3.1 Basic demographic characteristics

**Figure 1:**
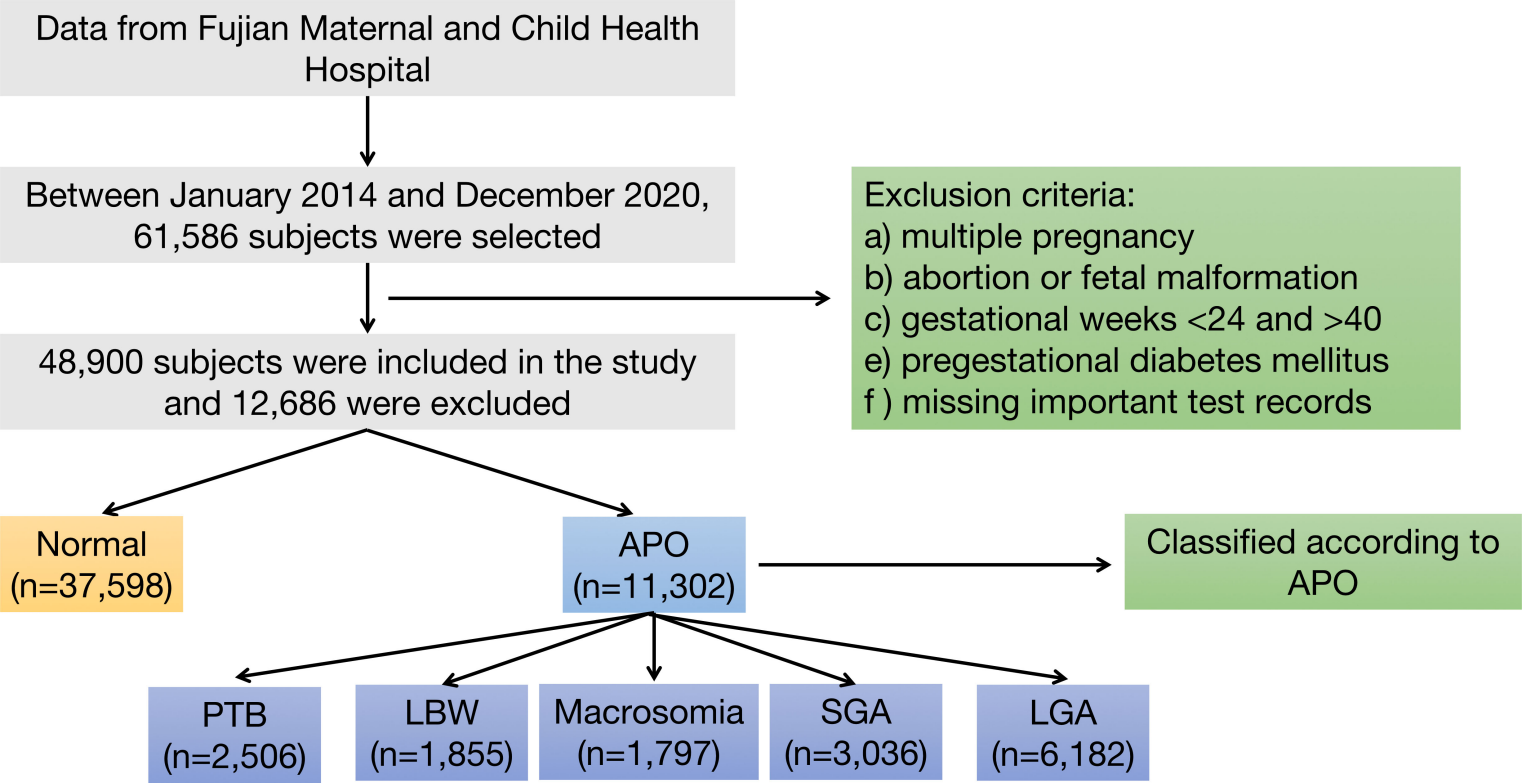
Flowchart for the inclusion of research subjects.

From January 1, 2014 to December 31, 2020, Fujian Maternal and Child Health Hospital followed 61,586 pregnant women. After excluding 12,686 participants who did not meet the study criteria, a final cohort of 48,900 eligible pregnant women was established. Within this cohort, 37,598 pregnant women’s fetuses without any type of APO were classified as the normal group. The remaining 11,302 pregnant women’s fetuses with APO formed the APO group, which was further subdivided into subgroups: PTB group, LBW group, macrosomia group, SGA group and LGA group. The complete research workflow is illustrated in **Figure 1**.

### 3.2 Clinical characteristics

As shown in **Table 1**, the average age of pregnant women in this study was 30.2 years, with a standard deviation of 4.4 years, and the ratio of male to female fetuses was about 1.17:1. Additionally, the proportion of elderly parturient women was 17.0%, while the rates of gestational hypertension and gestational thyroid disorders were 2.0% and 4.8%, respectively. Compared with the APO group, the normal group had younger, a lower rate of gestational hypertension, and a lower proportion of assisted reproduction (*P*<0.001).

**Table 1:** Characteristics of study subjects and their fetuses.

Variables	Total (n=48900)	Normal (n=37598)	APO						P
			All APO (n=11302)	PTB (n=2506)	LBW (n=1855)	Macrosomia (n=1797)	SGA (n=3036)	LGA (n=6182)	
Maternal age, year ( $\bar{x}\;\pm \;\text{s}$ )	30.2 ± 4.4	30.1 ± 4.3	30.6 ± 4.5	30.8 ± 4.6	30.5 ± 4.5	31.1 ± 4.6	29.5 ± 4.2	31.2 ± 4.6	**<0.001**
Elderly parturient, n (%)									**<0.001**
Yes	8304 (17.0)	6031 (16.0)	2273 (20.1)	539 (21.5)	348 (18.8)	413 (23.0)	368 (12.1)	1487 (24.1)	
No	40596 (83.0)	31567 (84.0)	9029 (79.9)	1967 (78.5)	1507 (81.2)	1384 (77.0)	2668 (87.9)	4695 (75.9)	
Gravidity, n (%)									**<0.001**
1	19724 (40.4)	15435 (41.1)	4289 (37.9)	963 (38.4)	837 (45.1)	542 (30.2)	1659 (54.6)	1783 (28.8)	
2	14981 (30.6)	11581 (30.8)	3400 (30.1)	767 (30.6)	508 (27.4)	578 (32.2)	771 (25.4)	1997 (32.3)	
≥3	14195 (29.0)	10582 (28.1)	3613 (32.0)	776 (31.0)	510 (27.5)	677 (37.6)	606 (20.0)	2402 (38.9)	
Parity, n (%)									**<0.001**
1	27307 (55.8)	21276 (56.6)	6031 (53.4)	1405 (56.1)	1165 (62.8)	805 (44.8)	2176 (71.7)	2646 (42.8)	
2	19945 (40.8)	15102 (40.2)	4843 (42.8)	1016 (40.5)	651 (35.1)	899 (50.0)	808 (26.6)	3228 (52.2)	
≥3	1648 (3.4)	1220 (3.2)	428 (3.8)	85 (3.4)	39 (2.1)	93 (5.2)	52 (1.7)	308 (5.0)	
Assisted reproduction, n (%)									**<0.001**
Yes	1044 (2.1)	754 (2.1)	290 (2.6)	80 (3.2)	51 (2.7)	56 (3.1)	57 (1.9)	167 (2.7)	
No	47856 (97.9)	36844 (97.9)	11012 (97.4)	2426 (96.8)	1804 (97.3)	1741 (96.9)	2979 (98.1)	6015 (97.3)	
Gestational hypertension, (%)									**<0.001**
Yes	964 (2.0)	695 (1.8)	269 (2.4)	81 (3.2)	72 (3.9)	34 (1.9)	91 (3.0)	117 (1.9)	
No	47936 (98.0)	36903 (98.2)	11033 (97.6)	2425 (96.8)	1783 (96.1)	1763 (98.1)	2945 (97.0)	6065 (98.1)	
Gestational thyroid disorders, n (%)									0.95
Yes	2361 (4.8)	1817 (4.8)	544 (4.8)	122 (4.9)	97 (5.2)	77 (4.3)	159 (5.2)	279 (4.5)	
No	46539 (95.2)	35781 (95.2)	10758 (95.2)	2384 (95.1)	1758 (94.8)	1720 (95.7)	2877 (94.8)	5903 (95.5)	
Delivery mode, n (%)									**<0.001**
Eutocia	32225 (65.9)	25700 (68.4)	6525 (57.7)	1411 (56.3)	976 (52.6)	834 (46.4)	2074 (68.3)	3256 (52.7)	
Cesarean	16675 (34.1)	11898 (31.6)	4777 (42.3)	1095 (43.7)	879 (47.4)	963 (53.6)	962 (31.7)	2926 (47.3)	
Infant sex, n (%)									0.52
Male	26348 (53.9)	20228 (53.8)	6120 (54.1)	1453 (58.0)	851 (45.9)	1236 (68.8)	1641 (54.1)	3326 (53.8)	
Female	22552 (46.1)	17370 (46.2)	5182 (45.9)	1053 (42.0)	1004 (54.1)	561 (31.2)	1395 (45.9)	2856 (46.2)	

APO, adverse pregnancy outcomes; PTB, preterm birth; LBW, low birth weight; SGA, small for gestational age; LGA, large for gestational age. *P* represents the comparison of the normal group (column 3) with all APO group (column 4), using either the Chi-square test or the Kruskal-Wallis rank sum test. Bold represents *P* values less than 0.05.

**Figure 2:**
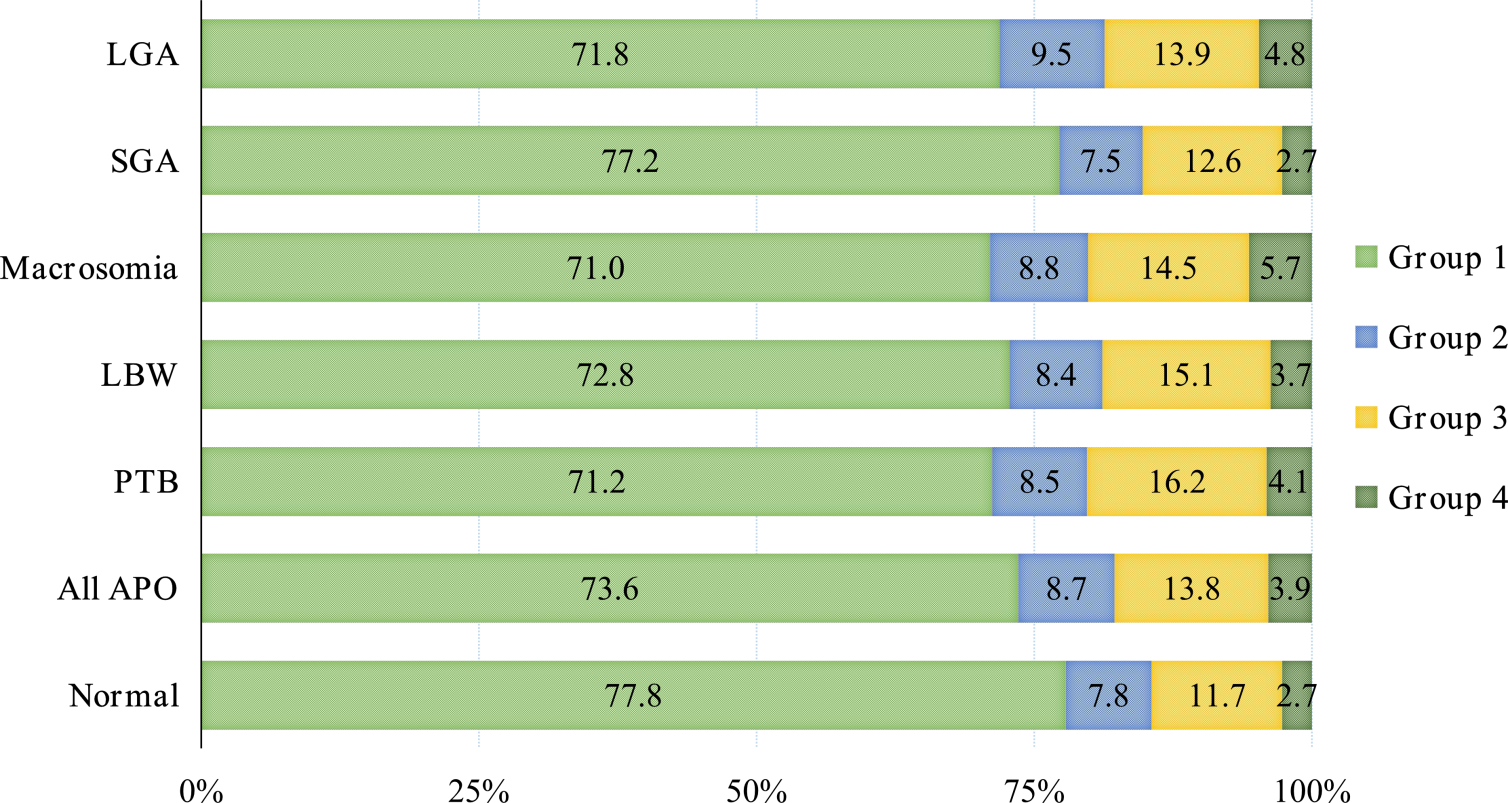
Plot of mid-pregnancy plasma glucose levels of subjects in different groups.

The APO group exhibited a higher prevalence of abnormal plasma glucose levels compared to the normal group. These abnormalities predominantly manifested as normal at 0h, but abnormal at 1h or 2h (Group 3), accounting for 13.8%. Among various APO subtypes, Group 3 represented the primary abnormal pattern, with proportions of 16.2% (PTB), 15.1% (LBW), 14.5% (macrosomia), 12.6% (SGA) and 13.9% (LGA), respectively, as shown in **Figure 2**.

### 3.3 Association between fetal APO and plasma glucose levels in mid-pregnancy

This study further conducted a univariate binary logistic regression analysis to assess the associations between confounding factors and APO. The model revealed that elderly parturient, delivery mode, parity, gravidity, gestational hypertension, and assisted reproduction were all significantly associated with APO. However, infant sex and gestational thyroid disorders showed no significant association with APO (as shown in **Table 2**).

**Table 2:** Binary logistic regression analysis of associations between covariates and adverse pregnancy outcomes.

Variable	OR (95%CI)	*P*
Elderly parturient		**<0.001**
No	1 (Ref)	
Yes	1.32 (1.25-1.39)	
Gravidity		
1	1 (Ref)	
2	1.06 (1.00-1.11)	**0.035**
≥3	1.23 (1.17-1.29)	**<0.001**
Parity		
1	1 (Ref)	
2	1.13 (1.08-1.18)	**<0.001**
≥3	1.24 (1.10-1.39)	**<0.001**
Assisted reproduction		**<0.001**
No	1 (Ref)	
Yes	1.29 (1.12-1.47)	
Gestational hypertension		**<0.001**
No	1 (Ref)	
Yes	1.29 (1.12-1.49)	
Gestational thyroid disorders		0.93
No	1 (Ref)	
Yes	0.99 (0.90-1.10)	
Delivery mode		**<0.001**
Eutocia	1 (Ref)	
Cesarean	1.58 (1.51-1.65)	
Infant sex		0.51
Male	1 (Ref)	
Female	0.99 (0.95-1.03)	

Bold represents *P* values less than 0.05.

**Table 3** presented the results of binary logistic regression analysis, which assessed the association between mid-pregnancy plasma glucose levels and APO. In the crude model, compared with the normal group, the risk of APO with GDM and abnormal plasma glucose levels at three time points (0h≥5.1mmol/L, 1h≥10.0mmol/L, 2h≥8.5mmol/L) increased (OR=1.26, 1.24, 1.36, 1.33, all *P*<0.001). Group 2, Group 3, and Group 4 all demonstrated significantly higher risk of APO compared to Group 1 (OR=1.18, 95%CI: 1.10-1.28; OR=1.24, 95%CI: 1.17-1.32; OR=1.56, 95%CI: 1.39-1.74, respectively). Additionally, there was a trend of increasing risk of APO with higher group numbers (*P*<0.001). After adjusting for maternal age, gravidity, parity, assisted reproduction, gestational hypertension, gestational thyroid disorders, delivery mode and infant sex, abnormal plasma glucose levels at three time points were associated with APO (all *P*<0.001). Similarly, Group 2, Group 3 and Group 4 still had a higher risk of APO compared to Group 1 (OR=1.14, 95%CI: 1.06-1.24; OR=1.18, 95%CI: 1.10-1.25; OR=1.42, 95%CI: 1.26-1.59, respectively), and it has an increasing trend (*P*<0.001).

**Table 3:** Binary logistic regression analysis of association between mid-pregnancy plasma glucose levels and adverse pregnancy outcomes.

Variable	n (%)	Crude		Adjusted	
		OR (95%CI)	*P*	OR (95%CI)	*P*
GDM			**<0.001**		**<0.001**
No	37562 (76.8)	1 (Ref)		1 (Ref)	
Yes	11338 (23.2)	1.26 (1.20-1.32)		1.19 (1.14-1.25)	
OGTT 0h			**<0.001**		**<0.001**
<5.1 mmol/L	43547 (89.1)	1 (Ref)		1 (Ref)	
≥5.1 mmol/L	5353 (10.9)	1.24 (1.16-1.32)		1.18 (1.11-1.26)	
OGTT 1h			**<0.001**		**<0.001**
<10 mmol/L	43626 (89.2)	1 (Ref)		1 (Ref)	
≥10 mmol/L	5274 (10.8)	1.36 (1.27-1.45)		1.28 (1.20-1.37)	
OGTT 2h			**<0.001**		**<0.001**
<8.5 mmol/L	44198 (90.4)	1 (Ref)		1 (Ref)	
≥8.5 mmol/L	4702 (9.6)	1.33 (1.24-1.42)		1.24 (1.16-1.33)	
OGTT groups					
Group 1	37562 (76.8)	1 (Ref)		1 (Ref)	
Group 2	3908 (8.0)	1.18 (1.10-1.28)	**<0.001**	1.14 (1.06-1.24)	**<0.001**
Group 3	5985 (12.2)	1.24 (1.17-1.32)	**<0.001**	1.18 (1.10-1.25)	**<0.001**
Group 4	1445 (3.0)	1.56 (1.39-1.74)	**<0.001**	1.42 (1.26-1.59)	**<0.001**
*P* for trend			**<0.001**		**<0.001**

GDM, gestational diabetes mellitus; OGTT, oral glucose tolerance test. Group 1 represents normal plasma glucose at 0h, 1h and 2h; Group 2 represents abnormal plasma glucose at 0h, but normal plasma glucose at 1h and 2h; Group 3 represents normal plasma glucose at 0h, but abnormal plasma glucose at 1h or 2h; Group 4 represents abnormal plasma glucose at 0h, 1h or 2h. Adjusted for maternal age, gravidity, parity, assisted reproduction, gestational hypertension, gestational thyroid disorders, delivery mode and infant sex. Bold represents *P* values less than 0.05.

### 3.4 Association between different types of fetal APO and plasma glucose levels in mid-pregnancy

**Table 4** showed the associations between mid-pregnancy plasma glucose levels and different types of APO. After adjusting for confounding factors, GDM, abnormal plasma glucose levels at three time points were significantly associated with macrosomia (OR=1.25, 95%CI:1.12-1.39; OR=1.29, 95%CI:1.13-1.48; OR=1.41, 95%CI:1.23-1.61; OR=1.23, 95%CI:1.06-1.43, respectively), and also showed positive associations with LGA (OR=1.23, 95%CI:1.15-1.31; OR=1.32, 95%CI:1.22-1.43; OR=1.28, 95%CI:1.18-1.39; OR=1.21, 95%CI:1.11-1.31, respectively). In addition, GDM, abnormal at 1h and 2h were significantly associated with PTB (OR=1.26, 95%CI:1.15-1.38; OR=1.37, 95%CI:1.22-1.54; OR=1.38, 95%CI:1.22-1.55, respectively), and also showed positive associations with LBW (OR=1.18, 95%CI:1.06-1.32; OR=1.25, 95%CI:1.09-1.43; OR=1.32, 95%CI:1.14-1.51, respectively), while abnormal at 2h was significantly associated with SGA (OR=1.16, 95%CI:1.02-1.31). The risks of PTB, LBW, macrosomia and LGA increased with an increase in the number of OGTT groups (*P*<0.001).

**Table 4:** Binary logistic regression analysis of association between mid-pregnancy plasma glucose levels and different types of adverse pregnancy outcomes.

Variable	PTB		LBW		Macrosomia		SGA		LGA	
Adjusted	OR (95%CI)	*P*	OR (95%CI)	*P*	OR (95%CI)	*P*	OR (95%CI)	*P*	OR (95%CI)	*P*
GDM		**<0.001**		**0.002**		**<0.001**		0.38		**<0.001**
No	1 (Ref)		1 (Ref)		1 (Ref)		1 (Ref)		1 (Ref)	
Yes	1.26 (1.15-1.38)		1.18 (1.06-1.32)		1.25 (1.12-1.39)		1.04 (0.95-1.14)		1.23 (1.15-1.31)	
OGTT 0 h		0.09		0.33		**<0.001**		0.64		**<0.001**
<5.1 mmol/L	1 (Ref)		1 (Ref)		1 (Ref)		1 (Ref)		1 (Ref)	
≥5.1 mmol/L	1.11 (0.98-1.26)		1.07 (0.93-1.24)		1.29 (1.13-1.48)		0.97 (0.86-1.10)		1.32 (1.22-1.43)	
OGTT 1 h		**<0.001**		**0.001**		**<0.001**		0.18		**<0.001**
<10 mmol/L	1 (Ref)		1 (Ref)		1 (Ref)		1 (Ref)		1 (Ref)	
≥10 mmol/L	1.37 (1.22-1.54)		1.25 (1.09-1.43)		1.41 (1.23-1.61)		1.09 (0.96-1.22)		1.28 (1.18-1.39)	
OGTT 2 h		**<0.001**		**<0.001**		**0.005**		**0.021**		**<0.001**
<8.5 mmol/L	1 (Ref)		1 (Ref)		1 (Ref)		1 (Ref)		1 (Ref)	
≥8.5 mmol/L	1.38 (1.22-1.55)		1.32 (1.14-1.51)		1.23 (1.06-1.43)		1.16 (1.02-1.31)		1.21 (1.11-1.31)	
OGTT groups										
Group 1	1 (Ref)		1 (Ref)		1 (Ref)		1 (Ref)		1 (Ref)	
Group 2	1.11 (0.96-1.28)	0.16	1.08 (0.91-1.27)	0.39	1.13 (0.95-1.34)	0.15	0.98 (0.85-1.12)	0.75	1.24 (1.12-1.36)	**<0.001**
Group 3	1.35 (1.20-1.51)	**<0.001**	1.25 (1.09-1.42)	**0.001**	1.18 (1.03-1.36)	**0.018**	1.09 (0.97-1.22)	0.13	1.13 (1.04-1.22)	**0.004**
Group 4	1.34 (1.08-1.64)	**0.007**	1.21 (0.94-1.55)	0.13	1.85 (1.49-2.28)	**<0.001**	1.00 (0.79-1.25)	0.97	1.64 (1.43-1.87)	**<0.001**
*P* for trend		**<0.001**		**<0.001**		**<0.001**		0.28		**<0.001**

GDM, gestational diabetes mellitus; OGTT, oral glucose tolerance test; PTB, preterm birth; LBW, low birth weight; SGA, small for gestational age; LGA, large for gestational age. Group 1 represents normal plasma glucose at 0h, 1h and 2h; Group 2 represents abnormal plasma glucose at 0h, but normal plasma glucose at 1h and 2h; Group 3 represents normal plasma glucose at 0h, but abnormal plasma glucose at 1h or 2h; Group 4 represents abnormal plasma glucose at 0h, 1h or 2h. Adjusted for maternal age, gravidity, parity, assisted reproduction, gestational hypertension, gestational thyroid disorders, delivery mode and infant sex. Bold represents *P* values less than 0.05.

### 3.5 Subgroup analysis

**Figure 3:**
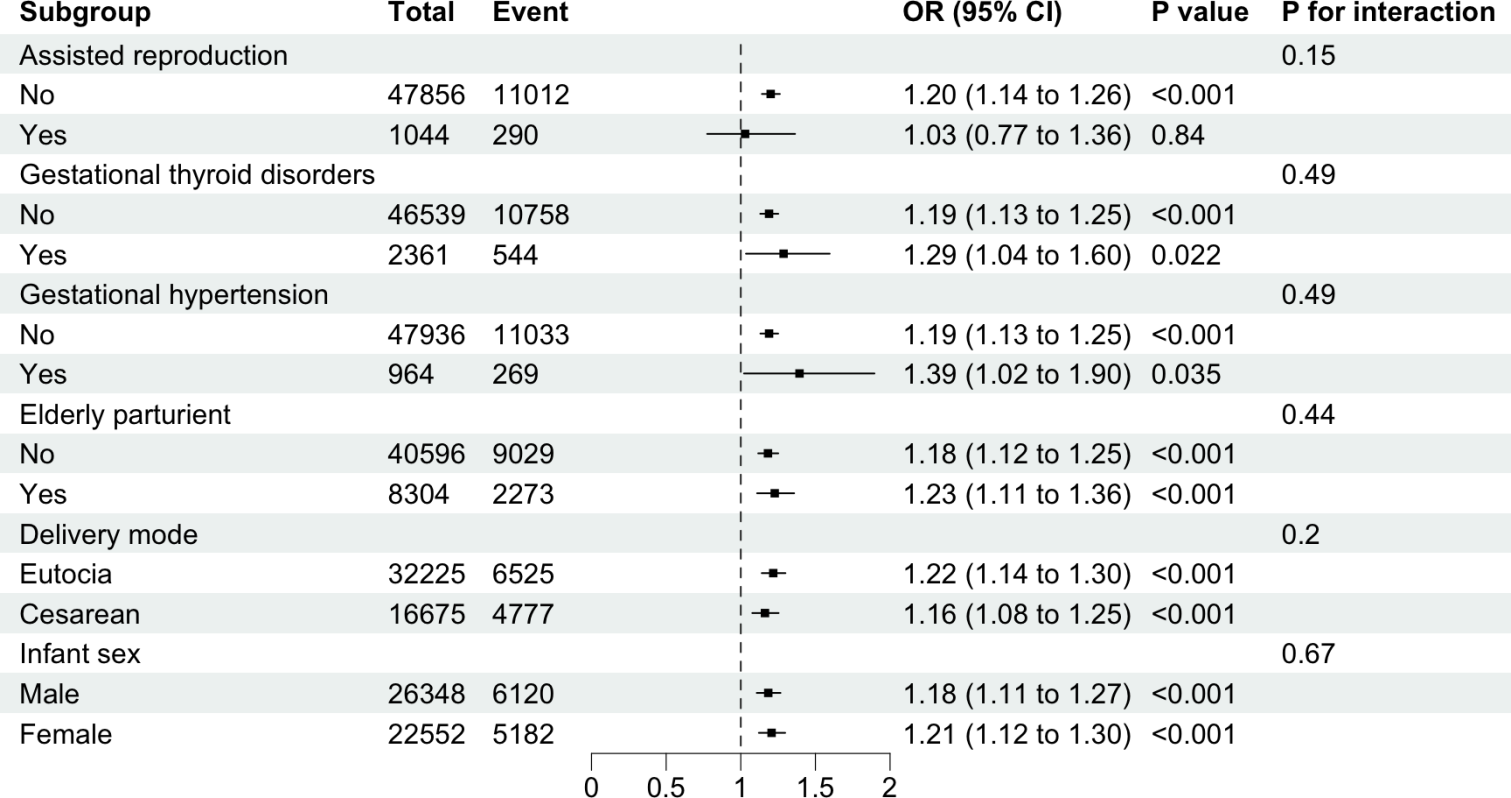
Subgroup analysis of the associations between gestational diabetes mellitus and risks of adverse pregnancy outcomes. Binary logistic regression adjusted for maternal age, gravidity, parity, assisted reproduction, gestational hypertension, gestational thyroid disorders, delivery mode, and infant sex.

The subgroup analysis revealed the potential association between GDM and APO across different subgroups, including gestational hypertension, gestational thyroid disorders, delivery mode, infant sex and elderly parturient (*P*<0.05, as shown in **Figure 3**). In the assisted reproduction subgroup, the non-assisted reproduction group showed statistical significance, while the assisted reproduction group did not demonstrate statistical significance. This difference was not observed in other subgroups. Interaction tests revealed that assisted reproduction, gestational thyroid disorders, gestational hypertension, delivery mode, infant sex and elderly parturient had no significant impact on this association (all *P*>0.05).

### 3.6 Sensitivity analysis

This study also conducted a sensitivity analysis using logistic regression. Since elderly parturient, assisted reproduction, gestational thyroid disorders, and gestational hypertension might serve as risk factors for APO, we excluded those participants, ultimately including 37,423 participants in sensitivity analysis. After adjusting for confounding factors, GDM, abnormal plasma glucose levels at three time points were significantly associated with APO (OR=1.17, 95%CI:1.10-1.24; OR=1.15, 95%CI:1.06-1.24; OR=1.33, 95%CI:1.23-1.44; OR=1.24, 95%CI:1.14-1.36, respectively, as shown in **Table 5**). Additionally, there was an increasing trend as the number of OGTT groups increased (*P*<0.001). When APO were further categorized, PTB, LBW, macrosomia, and LGA were significantly associated with GDM (OR=1.15, 95%CI:1.03-1.29; OR=1.15, 95%CI:1.01-1.31; OR=1.28, 95%CI:1.12-1.46; OR=1.23, 95%CI:1.14-1.33, respectively), and the risk increased as the number of OGTT groups rose. (*P*<0.05, as shown in **Table 6**). Therefore, the results of the sensitivity analysis indicated that the conclusions of this study were stable and reliable.

**Table 5:** Binary logistic regression analysis of association between mid-pregnancy plasma glucose levels and adverse pregnancy outcomes (excluded participants with elderly parturient, assisted reproduction, gestational hypertension, and gestational thyroid disorders).

Variable	n (%)	Crude		Adjusted	
		OR (95%CI)	*P*	OR (95%CI)	*P*
GDM			**<0.001**		**<0.001**
No	29805 (79.6)	1 (Ref)		1 (Ref)	
Yes	7618 (20.4)	1.20 (1.13-1.27)		1.17 (1.10-1.24)	
OGTT 0h			**<0.001**		**<0.001**
<5.1 mmol/L	33682 (90.0)	1 (Ref)		1 (Ref)	
≥5.1 mmol/L	3741 (10.0)	1.18 (1.09-1.28)		1.15 (1.06-1.24)	
OGTT 1h			**<0.001**		**<0.001**
<10 mmol/L	34067 (91.0)	1 (Ref)		1 (Ref)	
≥10 mmol/L	3356 (9.0)	1.37 (1.26-1.48)		1.33 (1.23-1.44)	
OGTT 2h			**<0.001**		**<0.001**
<8.5 mmol/L	34573 (92.4)	1 (Ref)		1 (Ref)	
≥8.5 mmol/L	2850 (7.6)	1.28 (1.17-1.39)		1.24 (1.14-1.36)	
OGTT groups					
Group 1	29805 (79.6)	1 (Ref)		1 (Ref)	
Group 2	2892 (7.7)	1.10 (1.00-1.20)	**0.048**	1.07 (0.98-1.17)	0.14
Group 3	3877 (10.4)	1.19 (1.10-1.29)	**<0.001**	1.17 (1.08-1.26)	**<0.001**
Group 4	849 (2.3)	1.61 (1.39-1.87)	**<0.001**	1.54 (1.32-1.78)	**<0.001**
*P* for trend			**<0.001**		**<0.001**

GDM, gestational diabetes mellitus; OGTT, oral glucose tolerance test. Group 1 represents normal plasma glucose at 0h, 1h and 2h; Group 2 represents abnormal plasma glucose at 0h, but normal plasma glucose at 1h and 2h; Group 3 represents normal plasma glucose at 0h, but abnormal plasma glucose at 1h or 2h; Group 4 represents abnormal plasma glucose at 0h, 1h or 2h. Adjusted for maternal age, gravidity, parity, delivery mode and infant sex. Bold represents *P* values less than 0.05.

**Table 6:** Binary logistic regression analysis of association between mid-pregnancy plasma glucose levels and different types of adverse pregnancy outcomes (excluded participants with elderly parturient, assisted reproduction, gestational hypertension, and gestational thyroid disorders).

Variable	PTB		LBW		Macrosomia		SGA		LGA	
Adjusted	OR (95%CI)	*P*	OR (95%CI)	*P*	OR (95%CI)	*P*	OR (95%CI)	*P*	OR (95%CI)	*P*
GDM		**0.016**		**0.038**		**<0.001**		0.36		**<0.001**
No	1 (Ref)		1 (Ref)		1 (Ref)		1 (Ref)		1 (Ref)	
Yes	1.15 (1.03-1.29)		1.15 (1.01-1.31)		1.28 (1.12-1.46)		1.05 (0.95-1.16)		1.23 (1.14-1.33)	
OGTT 0 h		0.53		0.41		**<0.001**		0.94		**<0.001**
<5.1 mmol/L	1 (Ref)		1 (Ref)		1 (Ref)		1 (Ref)		1 (Ref)	
≥5.1 mmol/L	1.05 (0.90-1.22)		1.08 (0.90-1.28)		1.37 (1.15-1.61)		1.01 (0.87-1.15)		1.26 (1.15-1.39)	
OGTT 1 h		**<0.001**		**0.001**		**<0.001**		0.50		**<0.001**
<10 mmol/L	1 (Ref)		1 (Ref)		1 (Ref)		1 (Ref)		1 (Ref)	
≥10 mmol/L	1.43 (1.23-1.65)		1.32 (1.11-1.56)		1.53 (1.29-1.80)		1.05 (0.91-1.21)		1.38 (1.24-1.52)	
OGTT 2 h		**0.001**		0.06		**0.006**		0.17		**<0.001**
<8.5 mmol/L	1 (Ref)		1 (Ref)		1 (Ref)		1 (Ref)		1 (Ref)	
≥8.5 mmol/L	1.30 (1.11-1.53)		1.20 (0.99-1.44)		1.31 (1.08-1.57)		1.11 (0.95-1.29)		1.29 (1.15-1.44)	
OGTT groups										
Group 1	1 (Ref)		1 (Ref)		1 (Ref)		1 (Ref)		1 (Ref)	
Group 2	0.93 (0.77-1.11)	0.44	1.04 (0.85-1.27)	0.69	1.12 (0.91-1.37)	0.27	1.05 (0.90-1.22)	0.54	1.15 (1.02-1.29)	**0.019**
Group 3	1.22 (1.05-1.41)	**0.008**	1.19 (1.00-1.41)	**0.042**	1.17 (0.97-1.39)	0.09	1.08 (0.94-1.23)	0.26	1.17 (1.06-1.30)	**0.002**
Group 4	1.60 (1.22-2.06)	**<0.001**	1.30 (0.93-1.77)	0.11	2.34 (1.78-3.03)	**<0.001**	0.90 (0.66-1.19)	0.47	1.81 (1.51-2.15)	**<0.001**
*P* for trend		**<0.001**		**0.015**		**<0.001**		0.53		**<0.001**

GDM, gestational diabetes mellitus; OGTT, oral glucose tolerance test; PTB, preterm birth; LBW, low birth weight; SGA, small for gestational age; LGA, large for gestational age. Group 1 represents normal plasma glucose at 0h, 1h and 2h; Group 2 represents abnormal plasma glucose at 0h, but normal plasma glucose at 1h and 2h; Group 3 represents normal plasma glucose at 0h, but abnormal plasma glucose at 1h or 2h; Group 4 represents abnormal plasma glucose at 0h, 1h or 2h. Adjusted for maternal age, gravidity, parity, delivery mode and infant sex. Bold represents *P* values less than 0.05.

## 4 Discussion

Our study found that in women without pregestational diabetes, abnormal plasma glucose at different time points was positively associated with APO. Compared to fasting plasma glucose (FPG, OGTT 0h), post-load plasma glucose (OGTT 1h and 2h) exhibited more significant associations with different type of APO. Sensitivity analyses confirmed the stability of these findings. To our knowledge, this is the first study to explore the associations between OGTT different time points and APO in a Chinese population without pregestational diabetes. Our study provides novel insights into the interpretation of APO by highlighting the abnormal plasma glucose at different time points in OGTT.

In this study, abnormal plasma glucose at different time points in OGTT was positively associated with APO, and the risk of APO increased as the number of groups increased. This indicated that abnormal plasma glucose during mid-pregnancy posed a hazard to fetal development, consistent with the findings from Elena et al. (17). The study by Yun et al. revealed that women with intermediate hyperglycemia in early pregnancy face an increased risk of adverse maternal-fetal outcomes (18). Their study classified APO into two categories: LGA and primary cesarean delivery, while focusing solely on the impact of FPG. In contrast, our study categorized APO into five different groups, emphasizing post-load plasma glucose and stratifying OGTT results. This approach further clarified that abnormal post-load plasma glucose was more strongly associated with the occurrence of APO. While a study has indicated that OGTT 1h is associated with a higher risk of APO, our study highlights the necessity of simultaneously considering OGTT 2h for comprehensive risk assessment (19). Additionally, abnormal OGTT results can indicate glucose metabolic disorders, reflecting insulin secretion impairment or β-cell dysfunction (20). Since IFG represents increased FPG, while IGT represents increased plasma glucose levels at OGTT 2h, their manifestations of insulin resistance and secretion differ. In IFG, impaired initial insulin response to oral glucose normalizes in the later phase (60 to 120min), combined with severe hepatic insulin resistance, lead to abnormal FPG (21, 22). In contrast, IGT is characterized by both early and late-phase insulin secretion defects alongside predominant skeletal muscle insulin resistance, resulting in prolonged hyperglycemia after glucose loading (22, 23).

PTB is a syndrome that occurs before 37 weeks of gestation, triggered by infection, inflammation, and uterine overdistension (24). The association between PTB and GDM remains inconclusive, with inconsistent findings across studies (25–27). Our study results indicate that FPG showed no association with PTB, whereas post-load plasma glucose was significantly associated with PTB. Interestingly, we observed that the risk of APO increased with the OGTT groups, reaching the highest risk when both FPG and post-load plasma glucose were abnormal. This may be because FPG primarily reflects basal metabolic status, while concurrent skeletal muscle insulin resistance leads to sustained maternal hyperglycemia, prolonging fetal exposure to elevated plasma glucose levels (22). Hyperglycemia-induced oxidative stress plays a critical role in the development of diabetes (28). Oxidative stress accelerates placental aging, particularly in intrauterine tissues (fetal membranes of the placenta), and this process further contributes to PTB (29, 30). Additionally, miRNAs may play a mediating role between GDM and PTB. When overexpressed in rats, miRNAs stimulate insulin secretion, leading to β-cell dysfunction (31). The clinical study revealed differential expression of 15 plasma miRNAs in pregnant women with PTB compared to control group, providing further clues supporting this hypothesis (32).

Our study revealed consistent findings between macrosomia and LGA. Abnormal plasma glucose levels at all three time points independently contributed to these outcomes. Notably, the risk increased significantly with multiple abnormal OGTT time points compared to a single abnormality, demonstrating a trend towards increased these outcomes risks with increasing OGTT groups. Some studies have confirmed our findings, demonstrating significant differences in fetal size between pregnancies with GDM and normal group, with 4.15 times higher risk of LGA in GDM (33–35). Freinkel proposed that excessive circulating maternal glucose crossing the placenta provides energy to the fetus, while stimulating insulin secretion from fetal β-cell, ultimately leading to excessive fetal growth and obesity (36, 37). Consequently, maternal plasma glucose is generally considered the most important factor affecting fetal birth weight for macrosomia (38). An experimental study in diabetic rat models revealed that maternal hyperglycemia disrupts fetal development, resulting in a higher proportion of both smaller and larger weight (39). However, we failed to replicate these findings, with no consistent associations observed between GDM and LBW or SGA. Notably, different evidence exists showing that lower FPG and OGTT 2h significantly associated with LBW compared to GDM (40). These discrepancies underscore the necessity for further investigation into the underlying mechanisms.

Previous studies have suggested that assisted reproduction may elevate the risk of APO (41, 42). Our logistic regression analysis identified assisted reproduction as a significant risk factor for APO. Interestingly, subgroup analyses revealed different findings: in the non-assisted reproduction group, GDM had a significant association with APO, whereas no association in the assisted reproduction group. We hypothesize that women undergoing assisted reproduction due to local cultural and traditional influences may have adhered more rigorously to medical advice, maintained healthier dietary habits, and sought earlier medical interventions for fetal abnormalities. These behavioral modifications may attenuate the association between GDM and APO in the assisted reproduction group. Further research is needed to validate this hypothesis.

There were some limitations in this study. Firstly, the population data was sourced from the Fujian province of China, which does not represent the entire country and may therefore only be indicative of the situation in Fujian. Secondly, the study population enrolled from 2014 to 2020 can only represent past conditions and fails to reflect current situation. Thirdly, our study was retrospective and based on maternal registration data from the Fujian Maternal and Child Health Hospital, which lacking detailed information on confounding factors, such as dietary habits, smoking, alcohol consumption, maternal obesity, body mass index, heart diseases, other chronic medical conditions in pregnancy, and whether pregnant women received insulin treatment. Finally, the molecular mechanisms underlying these associations still require further research.

## 5 Conclusions

In conclusion, our study focused on plasma glucose at different time points, which more comprehensively revealed the association between GDM and APO. Compared to women with abnormal FPG, abnormal post-load plasma glucose was associated with APO. The highest risk was observed when both FPG and post-load plasma glucose were abnormal. This study facilitates the early identification of high-risk pregnant women for APO, thereby improving pregnancy outcomes. Therefore, hospitals should prioritize timely intervention for women with abnormal post-load plasma glucose. Further large-scale prospective studies are needed to validate these conclusions.

## Data Availability

The raw data supporting the conclusions of this article will be made available by the authors, without undue reservation.
